# Distribution of Nontuberculous Mycobacteria strains

**DOI:** 10.1186/1476-0711-12-33

**Published:** 2013-11-21

**Authors:** Murat Gunaydin, Keramettin Yanik, Cafer Eroglu, Ahmet Sanic, Ismail Ceyhan, Zayre Erturan, Riza Durmaz

**Affiliations:** 1 Department of Medical Microbiology, Faculty of Medicine, Ondokuz Mayis University, Samsun, Turkey; 2 Qafqaz University, Baku, Azerbaijan; 3 Public Health Institution of Turkey, National Tuberculosis Reference Laboratory, Ankara, Turkey; 4 Department of Medical Microbiology, Faculty of Medicine, Istanbul University, Istanbul, Turkey; 5 Department of Medical Microbiology, Faculty of Medicine, Yildirim Beyazit University, Ankara, Turkey

**Keywords:** Nontuberculous Mycobacteria, NTM, Mycobacteria other than tuberculosis, MOTT

## Abstract

**Aim:**

Mycobacteria other than tuberculosis (MOTT) cause increasingly serious infections especially in immunosuppressive patients by direct transmission from the environment or after colonization. However, identification of these species is difficult because of the cost and difficulties in defining to species level. Identification and distribution of these species can help clinician in the choice of treatment.

**Materials and methods:**

A total of 90 MOTT strains obtained from four different centers were included in the study. These strains were identified by sequence analysis of *16S rRNA* and *Hsp65* genetic regions.

**Results:**

Accordingly, within the 90 MOTT strains, 17 different species were identified. In order of frequency, these species were *M. gordonea* (*n* = 21), *M. abscessus* (*n* = 13), *M. lentiflavum* (*n* = 9), *M. fortuitum* (*n* = 8), *M. intracellulare* (*n* = 6), *M. kumamotonense* (*n* = 6), *M. neoaurum* (*n* = 5), *M. chimaera* (*n* = 5), *M. alvei* (*n* = 5), *M. peregrinum* (*n* = 3), *M. canariasense* (*n* = 3), *M. flavescens* (*n* = 1), *M. mucogenicum* (*n* = 1), *M. chelona* (*n* = 1), *M. elephantis* (*n* = 1), *M. terrae* (*n* = 1) and *M. xenopi* (*n* = 1). Most frequently identified MOTT species according to the geographical origin were as follows: *M. abscessus* was the most common species either in Istanbul or Malatya regions (*n* = 6, *n* = 6, consequently). While *M. kumamotonense* was the most frequent species isolated from Ankara region (*n* = 6), M*. gordonea* was the most common for Samsun region (*n* = 14).

**Conclusion:**

Our study revealed that frequency of MOTT varies depending on the number of clinical samples and that frequency of these species were affected by the newly identified species as a result of the use of novel molecular methods. In conclusion, when establishing diagnosis and treatment methods, it is important to know that infections caused by unidentified MOTT species may vary according to the regions in Turkey. The results of the study showed that there were differences in the frequency of MOTT species in the different geographical regions of Turkey.

## Introduction

Genus *Mycobacterium* causes an ever-increasing number of serious infections especially in immunosuppressive patients via colonization and environmental contamination [1]. Some mycobacteria are potentially pathogenic for people. Especially in recent years, increased incidence of Acquired Immunodeficiency Syndrome (AIDS) and widespread use of chemotherapeutic drugs for interventional therapies and cancer treatment have further increased the prevalence and importance of Mycobacteria other than tuberculosis (MOTT) infections [2,3]. These bacteria, commonly found in the environment, may cause various infections [1]. Studies have shown that MOTT colonize in respiratory tracts of hospitalized and dialysis patients and cause local abscess and nosocomial outbreaks especially in patients receiving injection or intravenous treatment [2,3]. Since the equipments used in hospitals (endoscopes, devices in dialysis units, etc.) may contain infectious bacteria, they may cause various infections in patients receiving interventional treatment. Despite the measures taken, one may experience difficulties in controlling and protecting some species which are resistant to disinfectants [2-4]. MOTT which may cause serious infections including affecting various systems in immunosuppressive patients are difficult to treat. Antibiotic treatment of *M. abscessus* infection, one of the rapidly growing MOTT species, is often unsuccessful and the risk of death increases in patients who have not undergone surgery [2,4]. Therefore, detection and identification of *Mycobacterium* species and determining of their antibiotic sensitivity are necessary for the control of infections caused by MOTT species and for the determination of epidemiology and treatment regimens. But clinical and laboratory diagnosis of infections is relatively difficult and expensive [4-6]. Therefore, in the cases in which identification is not possible, considering the results of the MOTT species distribution may help clinicians in the selection of empirical therapy [3,5,6]. For this purpose, we aimed to identify various MOTT species isolated from four different centers in our country at species-level and to establish the most frequently isolated MOTT species.

## Materials and methods

Ninety strains isolated from clinical specimens and identified as MOTT at the same center between 2001 and 2008 were used in the study. Of the strains, 40 were identified in Samsun Ondokuz Mayis University (OMU) Medical Faculty, Mycobacteriology Reference Laboratory, 19 in Ankara Refik Saydam Hygiene Center, Mycobacteriology Reference Laboratory, 20 in Malatya Inonu University, Medical Faculty, Mycobacteriology Reference Laboratory and 11 in Istanbul University, Istanbul Medical Faculty, Mycobacteriology Reference Laboratory. Strains were initially typed via classical methods. Then, sequencing of the 16S rRNA and Hsp65 gene regions were assessed. Sequencing results were compared with the sequences of mycobacteria in GenBank and those displaying compatibility over 98% were accepted as species [7].

## Results

DNA sequence analysis of both gene regions of the 90 strains with respect to their frequencies were defined as follows: *M. gordonae* (21), *M. abscessus* (13), *M. lentiflavum* (9), *M. fortuitum* (8)*, M. intracellulare* (6), *M. kumamotonense* (6), *M. neoaurum* (5), *M. chimaera* (5), *M. alvei* (5)*, M. peregrinum* (3) and *M. canariasense* (3) and *M. flavescense* (1), *M. mucogenicum* (1), *M. chelona* (1), *M. elephantis* (1), *M. terrae* (1), *M. xenopi* (1).

**Figure 1:**
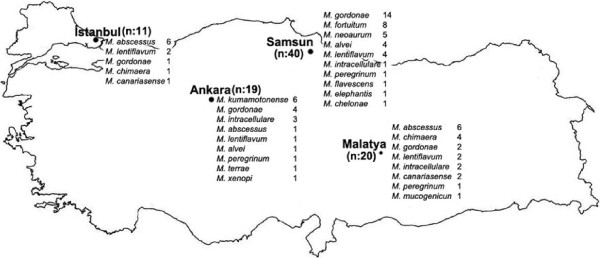
Distribution of nontuberculous mycobacteria strains in Turkey.

Considering the four strains from four centers, frequency order of the strains with respect to region was as follows: Of the strains obtained at OMU Medical Faculty, Mycobacteriology Laboratory (n = 40), the most frequently detected species were *M. gordonae* (14 strains), *M. fortuitum* (8 strains), *M. neoaurum* (5 strains), *M. lentiflavum* (4 strains), *M. alvei* (4 strains), *S. intracellulare* (1 strain), *M. peregrinum* (1 strain), *M. flavescense* (1 strain), *M. elephantis* (1 strain), *M. chelona* (1 strain); Of the strains obtained at Inonu University, Turgut Özal Medical Center, Mycobacteriology Laboratory (n = 20), the most frequently detected species were *M. abscessus* (6 strains), *M. chimaera* (4 strains), *M. gordonae* (2 strains), *M. canariasense* (2 strains), *M. lentiflavum* (2 strains), *M. intracellulare* (two strains), *M. peregrinum* (1 strain) *M. mucogenicum* (1 strain); Of the strains obtained at Istanbul University Medical Faculty, Mycobacteriology Laboratory (n = 11), the most frequently detected species were *M. abscessus* (6 strains) *M. lentiflavum* (2 strains), *M. canariasense* (2 strains), *M. chimaera* (1 strain) and of the strains obtained at Ankara Refik Saydam Hygiene Center Mycobacteria Laboratory (n = 19), the most frequently detected species were *M. kumamotonense* (6 strains), *M. gordonae* (4 strains), *M. intracellulare* (3 strains), *M. abscessus* (1 strain), *M. lentiflavum* (1 strain), *M. alvei* (1 strain), *M. peregrinum* (1 strain), *M. terrae* (1 strain), *M. xenopi* (1 strain) (Table 1). According to regions the distribution of MOTT species shown on the map (Figure 1).

**Table 1:** Distribution of MOTT strains with respect to centers they obtained

**Typing result** **n:90** **(100%)**	**Malatya** **n:20** **(22,3%)**	**Istanbul** **n:11** **(12,2%)**	**Samsun** **n:40** **(44,4%)**	**Ankara** **n:19** **(21,1%)**
***M. gordonae*** n:21 (23,3%)	2	1	14 (35%)*	4
***M. abscessus*** n:13 (14,4%)	6 (30%)*	6 (54,5%)*	-	1
***M. lentiflavum*** n:9 (10%)	2	2	4	1
***M. fortuitum*** n:8 (8,8%)	-	-	8	-
***M. intracellulare*** n:6 (6,6%)	2	-	1	3
***M. kumamotonense*** n:6 (6,6%)	-	-	-	6 (31,6%)*
***M. neoaurum*** n:5 (5,5%)	-	-	5	-
***M. alvei*** n:5 (5,5%)	-	-	4	1
***M. chimaera*** n:5 (5,5%)	4	1	-	-
***M. peregrinum*** n:3 (3,3%)	1	-	1	1
***M. canariasense*** n:3 (3,3%)	2	1	-	-
***M. flavescens*** n:1 (1,1%)	-	-	1	-
***M. mucogenicum*** n:1 (1,1%)	1	-	-	-
***M. terrae*** n:1 (1,1%)	-	-	-	1
***M. xenopi*** n:1 (1,1%)	-	-	-	1
***M. elephantis*** n:1 (1,1%)	-	-	1	-
***M. chelonae*** n:1 (1,1%)	-	-	1	-

n: Number of strains.

*The most commonly identified strains according to the provinces they obtained.

## Discussion

Atypical mycobacteria so-called MOTT may colonize and cause serious diseases especially in immunosuppressive patients as well as hospitalized and healthy people. Nontuberculous Mycobacterial factors constitute 0.5-35% of all mycobacterial infections in humans [8,9]. In patients with underlying disease such as AIDS, this rate approaches 50% [6]. Several studies have revealed that infections caused by these factors have become more important in recent years [4-6]. Studies also revealed that the number of hospital infections caused by MOTT have increased due to the reasons such as inadequate disinfection and sterilization. Progressive increases in the number of infections caused by MOTT, which are commonly found in our environment, make rapid and reliable identification of these species more important [3,4,10]. As the diagnosis and treatment of MOTT infections are mostly species-dependent and these strains are resistant to conventional anti-tuberculosis drugs, typing of *Mycobacterium* that requires different treatment protocols is necessary. However, typing of these species is not possible in every center [2,5,8]. Conventional methods used in typing of Mycobacteria are difficult to implement and molecular methods which are more sensitive and specific require more expensive materials. Their use is limited with difficulties encountered in standardization and requirement of specially trained personnel. This leads failures and delays in diagnosis and treatment [5,11,12]. Treatment of infections belonging to MOTT species which can not be identified due to insufficient infrastructures can be planned by considering the MOTT incidence of that region. Knowing frequency of nontuberculous mycobacterium species in our country will provide guidance on empirical treatments. But studies on this issue are limited in Turkey. In their study, Bedir et al. identified 11 *M. gordonae* from 17 MOTT species which were isolated from clinical specimens in Ankara province [9]. In their study conducted in Hacettepe University Hospital, Engin et al. found that *M. gordonae* (n = 11) was the most common one among 22 MOTT species isolated from clinical specimens [13]. Again, in their study conducted in Mersin University, Bayram et al. identified all MOTT species (n = 7) isolated from clinical specimens as *M. fortuitum*[14]. Çavuşoğlu et al. (2001) isolated 19 MOTT species from clinical specimens and found that *M. gordonae* was the most frequent one (57.9%) [15]. In their study conducted in Ege University, Çavuşoğlu et al. (2005) found that *M. fortuitum, M. peregrinum* and *M. intracellulare* were the most frequent ones among 29 MOTT species [16]. Uzun et al. identified 11 MOTT species and found that *M. fortuitum* was the most common species [17]. Bicmen et al. isolated 77 MOTT species and found that *M. gordonae* was the most frequently encountered species [18]. The incidence of MOTT infections is reported to increase with a rate of 8-9% each year [19]. While infections belonging to *Mycobacterium avium* complex (MAC), *M. xenopi* and *M. malmoense* species are prominent in Scandinavian countries and England, MAC species and *M. kansasii* infections in the United States and *M. malmoense* infections in Canada are the common cause of infections [2,6,20]. In Africa, *M. fortuitum* is the most frequent MOTT species isolated from the patients [6,19]. Studies regarding the incidence of MOTT in the world are numerous. Gruben-Jaworska et al. studied 4192 patients between 1999 and 2005 and identified 303 nontuberculous mycobacteria (NTM) [21]. According to the report of the Beijing Research Institute for Tuberculosis Control, of the 52 MOTT species 48.1% were identified as MAC and followed by *M. gordonae*[19]. In a comprehensive study conducted in Taiwan, *M. abscessus* and *M. avium* were found to be the most frequent species among MOTT which were isolated from patients and followed by other rapidly growing species [22]. In their study conducted in China, Hong-Xiu et al. found that *M. chelonae* was the most common species followed by *M. fortuitum* among 248 MOTT from clinical specimens [19]. Bold et al. suggested that slowly-growing species are usually isolated from lower respiratory tract whereas rapidly growing species are more frequently isolated from other tissues [23]. While MAC is the most frequently isolated MOTT species in West Asia, rapidly growing MOTT species are more frequent in Eastern Asia [24]. As can be seen in the studies, *M. avium, M. intracellulare, M. kansasii, M. fortuitum, M. abscessus* and *M. chelonae* are the most frequently encountered species in clinical practice [2,6,11,24-26]. *M. fortuitum, M. cholenae/absesus* and *M. smegmatis*, rapidly growing mycobacteria species of MOTT, may cause extrapulmonary disease and lead to hospital infections in comparison with slowly-growing species [3,6,12]. Unlike *M. fortuitum*, *M. abscessus* and *M. chelona* generally appear as cause of lower respiratory tract infection [3,6,22,24]. Although *M. gordonae* is the MOTT species that is most frequently isolated from environmental factors and contamination, it is important to be identified as a pathogen [6,27]. In a study conducted in Hong Kong, *M. gordonae* was reported to be the most frequent MOTT species isolated from clinical samples [26]. In a study conducted in England, incidence of MAC as well as *M. gordonae* infections have been reported to increase over the 10-year period [20]. Again, in a study conducted in Portugal, MAC was found to be the most frequently isolated species among 149 clinical specimens followed by *M. gordonae*[28]. In our study, of the 90 MOTT species obtained from four centers, 51 (60%) were rapidly growing MOTT species. According to the species-level identification, the most frequently detected MOTT species was *M. gordonae*, followed by *M. abscessus*, whereas *M. flavescense, M. mucogenicum, M. chelona, M. elephantis, M. terrae* and *M. xenopi* were rarely identified. In a study involving 50 centers regarding the regional distribution of species, MAC, *M. xenopi, M. kansasii, M. gordonae* and *M. fortuitum* have been reported to be the most commonly isolated species. It has also reported that while prevalence of MAC and *M. xenopi* has remarkably increased, the prevalence of *M. kansasii* and *M. gordonae* has decreased over the years. No significant change was observed in the isolation frequency of *M. fortuitum* and *M. chelonae* over the years [29]. *M. abscessus* is the most frequently isolated MOTT species in children [11]. It has been reported that *M. abscessus* is the leading cause lower respiratory tract infection among rapidly-growing species [2,19]. Although it shows an alteration depending on the region and the patient’s clinic picture, it is clear that isolation of rapidly-growing MOTT species from clinical samples is more common. At species-level, there are studies suggesting that *M. gordonae* is the most commonly isolated species [6,8,16,26,30]. But over the years, ever decreasing rate in the isolation of *M. gordonae* in comparison to other species has been observed [28]. This may due to the emerging measures not allowing contamination and colonization. In our study, *M. gordonae*, the mostly identified species from clinical specimens obtained from four centers, is consistent with the regional studies conducted in Turkey. Having abundant *M. gordonae* in the environment suggest that it may cause colonization and contamination of clinical samples [10,11,29,31]. Having low MAC frequency may be attributed to low AIDS incidence in our country and limited clinical samples and high *M. gordonae* colonization. Differences were observed in the frequency of MOTT species with respect to the region they obtained. While *M. abscessus* (6 strains) is the most frequently detected strain obtained from Istanbul (n = 11) and Malatya (n = 20), *M. kumamotonense* (6 strains) is the most frequently detected strain obtained from Ankara (n = 19). Of the strains obtained from Samsun (n = 40), *M. gordonae* (14 strains) is the mostly identified species. Having the result that *M. gordonae* is the most frequently identified strain in Samsun is consistent with that found in previous studies. It can be seen from the studies that incidence of *M. gordonae* is gradually increasing and it is frequently isolated from clinical specimens when MAC species which are commonly seen in AIDS infection are ignored [23,25,26]. Having MOTT species to be prominent in the identification of MOTT specimens obtained from Istanbul and Malatya is a novel knowledge for our country. The patients’ clinical picture, types of specimens and limited number of strains may have affected the high incidence of *M. abscessus*. It has been reported that this species is frequently isolated from tissue infections and it has a gradually increasing incidence as a cause of lower respiratory track and it frequently appears in nosocomial factors. It must also be noted that *M. gordonae* may have been a rare laboratory contaminant in these regions [3,8,11,22,23]. At the same time, it has been suggested that *M. abscessus* and analogous species have been isolated as infection agents in children rather than the other species [11]. However, we do not have knowledge about the patients so we could not make any comments on this issue. *M. kumamotonense,* the most commonly identified species obtained from Ankara, was identified in 2006 but *M. terra* which has a genotype displaying close similarity could not be discriminated. Therefore, as the studies on *M. kumamotonense* incidence are limited, it could have been identified in a different group [32,33]. We are of the opinion that prominent identification of this species in a region in Turkey is an important information.

As it can be seen in the studies, isolation frequency of MOTT species may differ according to the geographical region in which species were obtained as well as clinical material, clinical manifestation and underlying disease of the patient [2,4,6,19-32]. Since the treatment of lower respiratory tract infections caused by *M. abscessus* is difficult in comparison to other species, it is vital to consider this species in regions where this species were isolated [2,6,8,22].

MOTT species which are common in our environment may directly cause infections and also lead to various infections after many years of infection. For those centers which can not identify these species rapidly, knowing regional distribution of MOTT may be directive for the planning of treatment regimens. In our study, we observed that rapidly growing mycobacteria are more prominently isolated from samples throughout the country but at species-level *M. gordonae* was the most frequently isolated species followed by other rapidly growing species. We also found that MOTT frequency of four different centers may differ. Our study revealed that frequency of MOTT varies depending on the number of clinical samples and that frequency of these species were affected by the newly identified species as a result of the use of novel molecular methods. In conclusion, when establishing diagnosis and treatment methods, it is important to know that infections caused by unidentified MOTT species may vary according to the regions in Turkey.

## Competing interests

The authors declare that they have no competing interests.

## Authors’ contributions

MG is Project Manager, project designer and general supervisor, Laboratory working, writing the manuscript. KY carried out the all laboratory studies,and writing the manuscript. CE Laboratory supervisor, Analysis and interpretation of data and control of article. AS Project designer and general supervision of the research group. Old Project Manager *(He is worked in Samsun Ondokuz Mayis University, Now he is working Qafqaz University in Baku, Azerbajian)*. IC send strain for study from Ankara. ZE send strain for study from Istanbul and consultated of results. RD send strain for study from Malatya (He is worked in İnonu University in Malatya, Now he is working in Yildirm Beyazit University, in Ankara). All authors read and approved the final manuscript.

## References

[B1] Ingen JV Boeree MJ Dekhuijzen PNR Soolingen DV Environmental sources of rapid growing nontuberculous mycobacteria Clin Microbiol Infect 2009 15 888 893 19845700 10.1111/j.1469-0691.2009.03013.x

[B2] Jarzembowski JA Michael B Young MD Nontuberculous mycobacterial infections Arch Pathol Lab Med 2008 132 1333 1341 18684037 10.5858/2008-132-1333-NMI

[B3] Phillipsa MS Von Reyn CF Nosocomial Infections due to nontuberculous mycobacteria Clin Infect Dis 2001 33 1363 1374 11550115 10.1086/323126

[B4] De Groote MA Huitt G Infections due to rapidly growing mycobacteria Clin Infect Dis 2006 42 12 1756 1763 16705584 10.1086/504381

[B5] Wagner D Genç LS Nontuberculous mycobacterial infections: a clinical review Infection 2004 32 5 257 270 15624889 10.1007/s15010-004-4001-4

[B6] Falkinham JO III Epidemiology of infection by nontuberculous mycobacteria Clin Microbiol Rev 1996 9 2 177 215 8964035 10.1128/cmr.9.2.177 PMC172890

[B7] Turenne YC Tschetter L Wolfe J Kabani AA Necessity of quality-controlled 16S rRNA gene sequence databases: ıdentifying nontuberculous *mycobacterium* species J Clin Microbiol 2001 39 10 3637 3648 11574585 10.1128/JCM.39.10.3637-3648.2001 PMC88401

[B8] Koneman EW Allen SD Janda WM Color atlas and textbook of diagnostic microbiology 2006 5 Philadelphia: Lippincott-Raven Publishers 1064 1124

[B9] Bedir O Hasta örneklerinden izole edilen tüberküloz dışı mikobakterilerin moleküler yöntemlerle tanımlanması 2003 Gülhane Askeri Tıp Akademisi (Orhan Bedir, GATA, Dept of Clinical Microbiology, Dissertation. YÖK thesis no:131781)

[B10] Cafri U Aslan G Direkel Ş Tarhan G Ceyhan I Emektaş G Çevre Örneklerinden Mikobakteri Izolasyonu ve Tiplendirilmesi Mikrobiyol Bul 2010 44 395 403 21063989

[B11] Tortoli E Clinical manifestations of nontuberculous mycobacteria infections Clin Microbiol Infect 2009 15 906 910 19845702 10.1111/j.1469-0691.2009.03014.x

[B12] Ferdinand S Legrand E Goh KS Berchel M Mazzarelli G Sola C Tortoli E Rastogi N Taxonomic and phylogenetic status of non-tuberculous mycobacteria in Caribbean setting Mol Cell Probes 2004 18 6 399 408 15488380 10.1016/j.mcp.2004.06.006

[B13] Ergin MA Kocagöz T Us D Günalp A Polimeraz Zincir reaksiyonu-Restriksiyon Enzim Analizi ile Mikobakterilerin Tür Düzeyinde Tanımlanması Mikrobiyol Bült 1999 33 251 261

[B14] Bayram G Emektaş G Mikobakteri Türlerinin Idendifikasyonunda Polimerize Zincir Reaksiyonu-Parça Uzunluk Polimorfizmi Tekniği Ile Klasik Yöntemler Arasındaki Uyumun Belirlenmesi Mersin Üniversitesi Sağlık Bilim Dergisi 2008 1 3 8 13

[B15] Çavuşoğlu C Saydam CÇ Solak Ö Tuncel M Bilgiç A Klinik Örneklerden Izole edilen Tüberkuloz Dışı Mikobakterilerin Tanımlanması Mikrobiyol Bul 2001 35 1 69 75

[B16] Çavuşoğlu C Turhan A Yaygın YE Derici YK Bilgiç A Tüberkuloz Dışı Mikobakterileri Izolatlarının Tanımlanmasında Inno-Lipa Mycobacteria Inno-lipa Mycobacteria v2 ve Hsp65 dizi analiznin karşılaştırılamsı Mikrobiyol Bul 2005 39 4 437 445 16544545

[B17] Uzun M Erturan Z Anığ Ö Mycobacterium tuberculosis Kompleksi Suşlarının Erken Tanısında Kord Oluşumunun Değeri Klimik Dergisi 2000 13 1 27 29

[B18] Bicmen C Coskun M Gunduz AT Senol G Cirak AK Tibet G Nontuberculous mycobacteria isolated from pulmonary specimens between 2004 and 2009: causative agent or not? New Microbiol 2010 33 4 399 403 21213600

[B19] Hong-xiu W Jun Y Min H Jing-hui Y Rong-liang G Ling-jie J Shu-sheng Y Yan-lin Z Nontuberculous mycobacteria: susceptibility pattern and prevalence rate in Shanghai from 2005 to 2008 Chin Med J 2010 123 2 184 187 20137367

[B20] Moore JE Kruijshaar ME Ormerod LP Drobniewski F Abubakar I Increasing reports of non-tuberculous mycobacteria in England, Wales and Northern Ireland, 1995-2006 BMC Public Health 2010 10 612 20950421 10.1186/1471-2458-10-612 PMC2964631

[B21] Grubek-Jaworska H Walkiewicz R Safianowska A Nontuberculous mycobacterial infections among patients suspected of pulmonary tuberculosis Eur J Clin Microbiol Infect Dis 2009 28 739 744 19219472 10.1007/s10096-008-0694-0

[B22] Huang CT Tsai YJ Shu CC Lei YC Wang JY Yu CY Lee LN Yang PC Clinical significance of isolation of nontuberculous mycobacteria in pulmonary tuberculosis patients Respir Med 2009 103 1484 1491 19467850 10.1016/j.rmed.2009.04.017

[B23] Bolde EE Cunningham JA Della-Latta P Schluger NW Saiman L Epidemiology of nontuberculous mycobacteria in patients without HIV infection, New York City Emerg Infect Dis 2008 14 3 390 396 18325252 10.3201/eid1403.061143 PMC2570812

[B24] Simons S Lv I Hsueh PR Hung NV Dekhuijzen PN Boeree MJ Soolingen DV Nontuberculous mycobacteria in respiratory tract ınfections, Eastern Asia Emerg Infect Dis 2011 17 3 343 349 21392422 10.3201/eid1703100604 PMC3165997

[B25] Lessnau KD Milanee S Talavera W Mycobacterium gordonae: a treatable disease in HIV-positive patients Chest 1993 104 6 1779 1785 8252963 10.1378/chest.104.6.1779

[B26] Hosker HSR Lam CW Ng TK Ma HK Chan SL The prevalence and clinical significance of pulmonary infection due to non-tuberculous mycobacteria in Hong Kong Respir Med 1995 89 3 8 7708977 10.1016/0954-6111(95)90063-2

[B27] Tortoli E Phylogeny of the genus mycobacterium: many doubts, few certainties Infect Genet Evol 2012 12 4 827 831 21684354 10.1016/j.meegid.2011.05.025

[B28] Couto I Machado D Viveiros M Rodrigues L Amaral L Identification of nontuberculous mycobacteria in clinical samples using molecular methods: a 3-year study Clin Microbiol Infect 2010 16 8 1161 116 19832711 10.1111/j.1469-0691.2009.03076.x

[B29] Martín-Casabona N Bahrmand AR Bennedsen J Thomsen VO Curcio M Fauville-Dufaux M Feldman K Havelkova M Katila ML Köksalan K Pereira MF Rodrigues F Pfyffer GE Portaels F Urgell JR Rüsch-Gerdes S Tortoli E Vincent V Watt B Spanish Group for Non-Tuberculosis Mycobacteria: Non-tuberculous mycobacteria: patterns of isolation: a multi-country retrospective survey Int J Tuberc Lung Dis 2004 8 10 1186 1193 15527150

[B30] Yang SC Hsueh PR Lai HC High prevalence of antimicrobial resistance in rapidly growing mycobacteria in Taiwan Antimicrob Agents Chemother 2003 47 1958 1962 12760874 10.1128/AAC.47.6.1958-1962.2003 PMC155839

[B31] Cook JL Nontuberculous mycobacteria: opportunistic environmental pathogens for predisposed hosts Br Med Bull 2010 96 1 45 59 20977990 10.1093/bmb/ldq035

[B32] Tortoli E Impact of genotypic studies on mycobacterial taxonomy: the new mycobacteria of the 1990s Clin Microbiol Rev 2003 16 2 319 354 12692101 10.1128/CMR.16.2.319-354.2003 PMC153139

[B33] Masaki T Ohkusu K Hata H Fujiwara N Iihara H Yamada-Noda M Mycobacterium kumamotonense sp. nov. recovered from clinical specimen and the first isolation report of mycobacterium arupense in Japan: novel slowly growing, nonchromogenic clinical isolates related to mycobacterium terrae complex Microbiol Immunol 2006 50 889 897 17116985 10.1111/j.1348-0421.2006.tb03865.x

